# Association of Complement-Related Proteins in Subjects With and Without Second Trimester Gestational Diabetes

**DOI:** 10.3389/fendo.2021.641361

**Published:** 2021-03-30

**Authors:** Manjunath Ramanjaneya, Alexandra E. Butler, Meis Alkasem, Mohammed Bashir, Jayakumar Jerobin, Angela Godwin, Abu Saleh Md Moin, Lina Ahmed, Mohamed A. Elrayess, Steven C. Hunt, Stephen L. Atkin, Abdul-Badi Abou-Samra

**Affiliations:** ^1^ Qatar Metabolic Institute, Hamad Medical Corporation, Doha, Qatar; ^2^ Translational Research Institute, Hamad Medical Corporation, Doha, Qatar; ^3^ Diabetes Research Center (DRC), Qatar Biomedical Research Institute (QBRI), Hamad Bin Khalifa University (HBKU), Qatar Foundation (QF), Doha, Qatar; ^4^ Department of Laboratory Medicine and Pathology, Qatar Rehabilitation Institute, Hamad Medical Corporation, Doha, Qatar; ^5^ Department of Genetic Medicine, Weill Cornell Medicine-Qatar, Doha, Qatar; ^6^ Biomedical Research Center (BRC), Qatar University, Doha, Qatar; ^7^ Post Graduate Studies and Research, Royal College of Surgeons in Ireland Bahrain, Adliya, Bahrain

**Keywords:** complement, pregnancy, gestational diabetes, type 2 diabetes, preterm delivery

## Abstract

**Introduction:**

Gestational Diabetes Mellitus (GDM) development is related to underlying metabolic syndrome that is associated with elevated complement C3 and C4. Elevated C3 levels have been associated with preeclampsia and the development of macrosomia.

**Methods:**

This case-control study included 34 pregnant women with GDM and 16 non-diabetic (ND) women in their second trimester. Complement-related proteins were measured and correlated with demographic, biochemical, and pregnancy outcome data.

**Results:**

GDM women were older with a higher BMI (p<0.001); complement C3, C4 and Factor-H were significantly elevated (p=0.001, p=0.05, p=0.01, respectively). When adjusted for age and BMI, Complement C3 (p=0.04) and Factor-H (p=0.04) remained significant. Partial correlation showed significant correlation between C4 with serum alanine aminotransferase (ALT) (p<0.05) and 2^nd^ term diastolic blood pressure (p<0.05); Factor-H and C-reactive protein (CRP; p<0.05). Pearson bivariate analysis revealed significant correlations between C3, C4, and Factor-H and CRP; p<0.05; C3 and gestational age at delivery (GA; p<0.05); C4 and ALT and second-trimester systolic blood pressure (STBP) (p=0.008 and p<0.05, respectively); Factor-H and glycated hemoglobin (HbA1c) (p<0.05). Regression analysis showed that the elevation of C3 could be accounted for by age, BMI, GA and CRP, with CRP being the most important predictor (p=0.02). C4 elevation could be accounted for by ALT, CRP and STBP. CRP predicted Factor-H elevation.

**Conclusion:**

The increased C3, C4 and Factor-H during the second trimester of pregnancy in GDM are not independently associated with GDM; inflammation and high BMI may be responsible for their elevation. The elevation of second trimester C3 in GDM is associated with earlier delivery and further work is needed to determine if this is predictive.

## Introduction

Gestational diabetes (GDM) is the most frequent pregnancy-associated metabolic disorder, occurring in 14% of pregnant women (1, 2). GDM is usually identified in the latter stage of the second trimester (3). GDM is associated with increased risk of complications for both the mother and the fetus, including preterm delivery, cesarean delivery, preeclampsia, macrosomia, shoulder dystocia, neonatal hypoglycaemia and respiratory distress syndrome (4, 5). Women with a history of GDM are at increased risk for cardiovascular disease (6) and type 2 diabetes (T2DM) (7, 8) later in life.

During pregnancy, the nutritional demands of the fetus cause a physiological increase in insulin resistance in the mother (9). In a healthy pregnancy, compensatory mechanisms cause an increase in glucose-stimulated insulin release (10) together with an adaptive increase in beta-cell mass (11) to counterbalance the increase in insulin resistance. However, in pregnant women who are overweight or obese, insulin requirements are increased and, if the demand exceeds the insulin-secretory capacity, these conditions can increase risk for GDM (12). Complex, and not yet fully elucidated, mechanisms drive the pregnancy-related insulin resistance; notable factors are placental hormones, obesity, inactivity, poor diet, and genetics/epigenetics (13).

Older maternal age, pre-gravid overweight or obese status (14, 15), multiparity, ethnicity, a family history of diabetes, and excessive gestational weight gain (16) are recognized risk factors for the development of GDM.

Biomarkers that could predict GDM and its complications earlier in pregnancy than is currently possible using the standard oral glucose tolerance test would offer practical benefit for patient care and may provide a deeper understanding of the biochemical pathways involved in GDM and whether they parallel, or are divergent from, those in T2DM.

**Figure 1:**
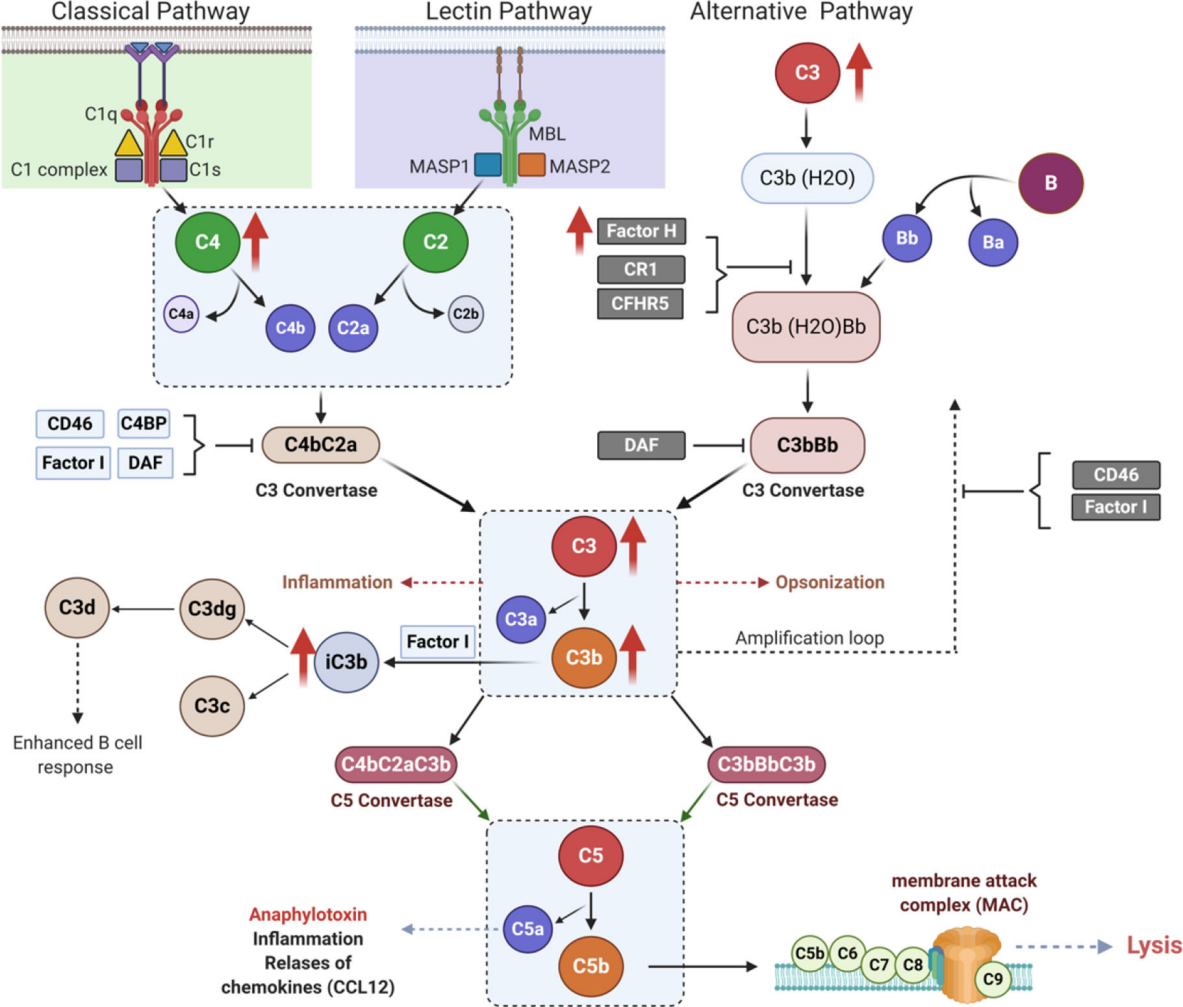
Complement activation pathways. Schematic illustration of classical (left panel), lectin (middle panel), and alternative (right panel) pathways of complement activation, showing links between initiator molecules, proteases and C3, C5 convertases. Upward red arrows indicate the complements that are elevated in the second trimester of pregnancy in gestational diabetes (GDM).

Pregnancy presents a unique immunologic state that initiates in earnest at implantation and usually resolves after delivery. Similarly, GDM is a unique metabolic state that begins and ends with gestation. The immunological changes that occur during normal and in GDM women during pregnancy is not clearly understood. The evolutionarily conserved complement system protects the host against bacterial infection, the individual elements forming a serine-protease cascade that attacks the membranes of invading organisms and induces cell lysis; hence, this system has been most studied in the context of infectious diseases (**Figure 1**). Though less well defined, the complement system also plays a role in metabolic disorders, and is essential for homeostasis of host cells, removal of apoptotic cells and priming of the adaptive immune response (17), processes which all occur in normal pregnancy and which may be dysregulated in pregnancies accompanied by metabolic disorders, such as GDM, and preeclampsia (17).

In normal pregnancy, increased activation of the complement system occurs with elevated plasma concentrations of C3a, C4a, and C5a (18), which may be important to counterbalance the normal suppression of adaptive immunity during pregnancy. Regulatory T cells enable fetal tolerance and complement activation controls T cell development (19, 20). Notably, preeclampsia has a well-known association with complement activation (21–25) though which regulatory T cells are decreased (26, 27). Factor D concentrations are increased (22). Complement activation, specifically factors C3, C4, C3a, Factor-H and Properdin, are associated with increased incident metabolic syndrome (28); and complement activation was related to adverse pregnancy outcomes, such as intrauterine growth retardation and GDM (24, 29). Further, polymorphisms of the mannose-binding lectin gene are associated with a higher risk for GDM (21). At term, patients with GDM have lower levels of C3a, C4a and C5a (30).

Given the elevation of complement in normal pregnancy we hypothesized that there would be further dysregulation of the complement system in GDM. Therefore, in this study, we sought to determine the serum complement protein concentrations in second trimester pregnant women with and without GDM and their relationship to patient demographics, biochemical parameters and pregnancy outcomes.

## Materials and Methods

### Study Design

This was a case-control study in 50 pregnant women (34 GDM and 16 non-diabetic (ND)) who were recruited during their second trimester at the antenatal clinic at the Women Wellness and Research Centre (WWRC) of Hamad Medical Corporation (HMC), Doha, Qatar, during 2016-2017. The study protocols were approved by the Institutional Review Boards (IRBs) of HMC (15101/15) and Weill Cornell Medical College in Qatar (WCMQ) (15-00016). Pregnant women between the ages of 18 and 40 without any previous medical history of chronic disease, in the second trimester of pregnancy and willing to comply with all study procedures and be available for the duration of the study were included. Pregnant women who are unable to provide informed consent, who were in first or third trimester of pregnancy, or currently enrolled in other clinical trials were excluded.

Demographics, anthropometrics, and medical history data were collected, including age, ethnicity, socio-economic background, vital signs, height, weight, menstrual cycle, the period of infertility, medications, complications, comorbidities, and family medical history. According to Qatar national guidelines for GDM, all pregnant women are screened at the first antenatal care visit by measuring fasting blood glucose (FBG). If FBG at the first visit was >5.1 mmol/l (92 mg/dl), 75 g OGTT is performed at 24 weeks’ gestation. The WHO criteria [FBG ≥5.1 mmol/L (92 mg/dl), 1-h post OGTT ≥10.0 mmol/L (180 mg/dl) or 2-h post OGTT ≥8.5 mmol/L (153 mg/dl)] were used for GDM diagnosis. GDM patients were seen back in the outpatient clinic within 1 week of the laboratory diagnosis of GDM. During this time blood samples were collected for complement proteins measurement. GDM patients were started on a diet for two weeks to achieve an FBG ≤ 5.3 mmol/l (95 mg/dl) and a 2-h post-prandial glucose ≤ 6.8 mmol/l (120 mg/dl) in ≥ 80% of the readings. If more than 20% of the readings were above targets, then metformin therapy was initiated and increased incrementally, followed by insulin supplementation if glucose targets were not achieved.

### Collection and Analysis of Blood Samples

Blood samples were collected and immediately processed and stored frozen at −80°C pending analysis, as previously reported (31). C reactive protein (CRP) was measured using magnetic bead based multiplex assay (BIO-RAD, Hertfordshire, U.K detection range 12 – 0.013 ng/ml, intra-assay CV 4% and inter-assay CV 6%). Analytical measurements for lipid parameters were total cholesterol (reference range: <5.17 mmol/L desirable; 5.17–6.18 mmol/L borderline high; >6.18 mmol/L high; clinical reportable range: 0.16 – 72 mmol/L CV 0.8%), triglycerides (reference range; normal <1.70 mmol/L; borderline high 1.70 – 2.2 mmol/L; high 2.2 -5.6 mmol/L; >5.6 mmol/L very high; clinical reportable range: 0.07 – 100 mmol/L CV 1.6%), and high-density lipoprotein cholesterol (HDL-C) (reference range above 1.0 mmol/L; clinical reportable range: 0.13 – 6.2 mmol/L; CV 2.6%) levels were measured using an ARCHITECT c Systems (Abbott Laboratories Sittingbourne, U.K) and measured in the Chemistry Laboratory at Hamad Medical Corporation, Doha, Qatar, using the manufacturer’s recommended protocol. Low-density lipoprotein cholesterol (LDL-C) (reference range: desirable < 3.36 mmol/L; borderline 3.36–4.11 mmol/L; high risk > 4.12 mmol/L; clinical reportable range 0.03 – 50 mmol/L; CV 2.1%) was calculated using the Friedewald equation. The analytical measurement range for ALT (reference range 0 – 55; clinical reportable range 5–4,700 U/L; CV 1.4%) and AST (reference range 5–34 U/L; clinical reportable range 2–4,565; CV 1%). HbA1c (4.2–20.1%) was determined by enzymatic method on ARCHITECT c Systems (clinical reportable range 4.2–20.1%; normal range: up to 5.6%, impaired glucose: 5.7–6.4%, diabetes: ≥ 6.5%; Abbott Laboratories Sittingbourne, U.K). Serum insulin was assayed using a competitive chemiluminescent immunoassay performed on the manufacturer’s DPC Immulite 2000 analyzer (Euro/DPC, Llanberis, UK). The analytical sensitivity of the insulin assay was 2 μU/ml, the coefficient of variation was 6%, and there was no stated cross-reactivity with proinsulin. Plasma glucose was measured using a Synchron LX 20 analyzer (Beckman-Coulter), using the manufacturer’s recommended protocol. The coefficient of variation for the assay was 1.2% at a mean glucose value of 5.3 mmol/L during the study period.

All patients gave written informed consent, and the conduct of the study was in accordance with ICH GCP and the Declaration of Helsinki. Pregnancy outcomes of gestational age at delivery, birth weight, maternal weight, blood pressure, and fetal outcome were recorded and collated with the apolipoprotein profile for all subjects who participated in the study.

### Human Complement Related Protein Measurements

Complement proteins were determined using the MILLIPLEX MAP Kit Human Complement Magnetic Bead Panel 1 (Cat # HCMP1MAG-19K) and MILLIPLEX MAP Kit Human Complement Magnetic Bead Panel 2 (Cat # HCMP2MAG-19K, Merck Millipore, USA). Milliplex kits are sensitive magnetic bead-based multiplexing protein panels that measure quantitative levels of these proteins simultaneously in serum samples. Complement protein levels in the samples were quantitated by the 5PL (five parameters) logistic regression algorithms that are built into the Bioplex manager six software, which were used for quantification of all serum samples in reference to standards. All the protein measurements were run on a Bioplex-200 (BIO-RAD, Hertfordshire, U.K.) instrument. The serum samples were diluted 200 times for complement panel 1 panel protein (C2, C4b, C5, C5a, C9, Factor D, Mannose-Binding Lectin and Factor I) and 40,000 times for complement panel 2 (C1q, C3, C3b/iC3b, C4, Factor B, Factor H and Properdin) to the protein levels within the reference range of the standard curve. The working range and assay precision for different Complement proteins were as follows C9 (41.2–30,000 ng/ml, intra-assay CV <10% and intra-assay CV <10%), Factor D (0.069–50 ng/ml, intra-assay CV <10% and intra-assay CV <10%), Mannose-Binding Lectin (0.137–100 ng/ml, intra-assay CV <10% and intra-assay CV <10%), Factor I (0.69–500 ng/ml, intra-assay CV <10% and intra-assay CV <10%), C2 (1.37–1,000 ng/ml, intra-assay CV <10% and intra-assay CV <10%), C4b (1.37 1000 ng/ml, intra-assay CV <10% and intra-assay CV <10%), C5 (2.74–2,000 ng/ml, intra-assay CV <10% and intra-assay CV <10%), C5a (4.12–3,000 ng/ml, intra-assay CV <10% and intra-assay CV <20%), C1q (0.08–60 ng/ml, intra-assay CV <10% and intra-assay CV <10%), C3 (0.27–200 ng/ml, intra-assay CV <10% and intra-assay CV <20%), C3b/iC3b (8.2–6,000 ng/ml, intra-assay CV <10% and intra-assay CV <20%), C4 (0.55–400 ng/ml, intra-assay CV <10% and intra-assay CV <10%), Factor B (0.08–60 ng/ml, intra-assay CV <10% and intra-assay CV <10%), Factor H (0.41–300 ng/ml, intra-assay CV <10% and intra-assay CV <10%) and Properdin (0.013–10 ng/ml, intra-assay CV <10% and intra-assay CV <20%).

### Statistical Analysis

There was no specific study on second trimester complement levels on which to power the study; however, given that GDM has many features of the metabolic syndrome, C3 differences between those with and without metabolic syndrome were used (28). An alpha of 0.05 with 80% power gave an effect size of 1.07, requiring a minimum of 15 subjects per group. (nQuery, Statsol USA). Descriptive statistics and means ± standard deviations (SD) were calculated for all continuous variables in the study. A general linear model was used to compare mean differences between control and GDM groups before and after adjustment for age and BMI. Pearson and partial correlations were performed to understand the associations between complement variables and demographic variables, with the partial correlations adjusted for age and BMI. Linear regression analysis was performed to determine predictors for circulating C3, C4 and Factor-H. All statistical analysis was done using statistical analysis SAS version 9.4 software. A statistical significance level (P-value) of <0.05 was considered as significant.

## Results

### Demographic and Biochemical Characteristics of Study Participants

Compared to the ND women, the GDM women were older (34.3 ± 4.4 vs 29.7 ± 4.2 years, p=0.001) and had a higher BMI (35.3 ± 5.6 vs 28.4 ± 6.4 kg/m^2^, GDM vs ND, p=0.0003). Baseline systolic and diastolic blood pressure were comparable between the 2 groups. GDM was diagnosed at 22.2 ± 4.1 weeks, and the control women were matched for gestational age (p=0.308). In keeping with the diagnosis, fasting plasma glucose was elevated in the GDM women (5.5 ± 0.9 vs 4.7 ± 0.3, GDM vs ND, p=0.039), though insulin and HbA1c did not differ between groups, indicative of the recent onset of hyperglycemia in the GDM women. Lipids (cholesterol, triglycerides, high- and low-density lipoproteins) and CRP did not differ between the cohorts, while ALT was elevated in the GDM women (17.5 ± 11.8 vs 10.2 ± 3.6 U/l, GDM vs ND, p=0.005).There were two premature deliveries in the control and five in the GDM group and no infant was LGA in our cohort. Gestational age at delivery and baby weight were similar between the cohorts (**Table 1**).

**Table 1:** Demographic and biochemical data of the gestational diabetes (GDM) and control women.

	Control	Sample size	GDM	Sample size	Student t-test
	Mean (SD)		Mean (SD)		p-value
Age (years)	29.7 (4.2)	16	34.3 (4.4)	34	0.001
BMI (kg/m2)	28.4 (6.4)	16	35.3 (5.6)	34	0.0003
Systolic-BP baseline (mmHg)	108.0 (12.0)	16	114.0 (12.0)	34	0.08
Diastolic-BP baseline (mmHg)	66.0 (8.0)	16	62.0 (6.0)	34	0.075
GDM diagnosis/gestational age (weeks)	20.8 (5.2)	16	22.2 (4.1)	34	0.308
Plasma glucose (mmol/L)	4.7 (0.3)	5	5.5 (0.9)	25	0.039
Insulin (uIU/L)	0.3 (0.3)	16	0.7 (1.1)	33	0.158
Glycated hemoglobin (HbA1c) (%)	5.0 (0.4)	13	5.2 (0.3)	33	0.081
Alanine aminotransferase (U/l)	10.2 (3.6)	16	17.5 (11.8)	28	0.005
Aspartate transaminase (U/l)	15.0 (3.6)	16	18.6 (8.4)	29	0.055
Cholesterol (mmol/L)	5.2 (1.3)	11	4.9 (1.2)	19	0.501
Triglycerides (mmol/L)	1.2 (0.7)	11	1. 5 (0.9)	19	0.425
High density lipoprotein (HDL) (mmol/L)	1.6 (0.4)	11	1.3 (0.3)	19	0.051
Low density lipoprotein (LDL)(mmol/L)	3.1 (1.0)	11	2.9 (0.9)	19	0.69
C-reactive protein (CRP) (mg/l)	88.0 (86.0)	16	123 (77)	31	0.177
2nd Term Systolic Blood Pressure (mmHg)	119.0 (11.0)	16	119.0 (9.0)	33	0.837
2nd Term Diastolic Blood Pressure (mmHg)	73.0 (6.0)	16	71.0 (8.0)	33	0.571
Weight at delivery (kg)	78.2 (12.9)	16	87.4 (14.0)	33	0.036
Gestational Age at delivery (weeks)	38.4 (1.6)	16	37.9 (1.5)	33	0.341
Baby Weight (grams)	2973.0 (508.0)	16	2991.0 (589.0)	31	0.92
Premature delivery	2	16	5	32	
LGA babies*	0	16	0	32	

*LGA, large for gestational age; 90^th^ percentile for age and was taken as 4000g.

### Complement Proteins

Complement C3 (279 ± 126 vs 162 ± 56 µg/ml, GDM vs ND, p=0.001), complement C4 (550 ± 96 vs 487 ± 105 µg/ml, GDM vs ND, p=0.05) and Factor-H (358 ± 57 vs 310 ± 66 µg/ml, GDM vs ND, p=0.01) were all elevated in the GDM women compared with ND women. Complement C1q, Factor-B, Properdin and the Anti-complement proteins were not different between the two cohorts (**Table 2**). After adjustment for age and BMI, only complement C3 (p=0.04) and Factor-H (p=0.04) remained significantly different between GDM and ND women.

**Table 2:** Complement and anti-complement levels in gestational diabetes (GDM) and control women.

	Control (n = 16)	GDM (n = 34)	Unadjusted	Adjusted
	Mean (SD)	Mean (SD)	p-value	p-value
Anti-Complement C2 (µg/ml)	6.9 (3.4)	7.1 (4.6)	0.89	0.29
Anti-Complement C4b (µg/ml)	12.8 (3.1)	13.3 (2.9)	0.64	0.28
Anti-Complement C5 (µg/ml)	41.2 (6.1)	40.3 (5.8)	0.62	0.91
Anti-Complement C5a (µg/ml)	0.7 (0.5)	0.8 (0.5)	0.82	0.45
Anti-Complement Factor-D (µg/ml)	2267.0 (403.0)	2148.0 (404.0)	0.33	0.22
Anti-Mannose-binding lectin (µg/ml)	2.9 (2.7)	2.5 (2.6)	0.62	0.63
Anti-complement factor-1 (µg/ml)	36.1 (6.9)	37.0 (9.7)	0.5	0.33
Complement- C1q (µg/ml)	82.4 (18.9)	80.0 (13.9)	0.63	0.62
Complement C3 (µg/ml)	162.0 (56.0)	279.0 (126.0)	0.001	0.04
Complement C3b/iC3b (µg/ml)	373.0 (270.0)	666.0 (561.0)	0.06	0.12
Complement C4 (µg/ml)	487.0 (105.0)	550.0 (96.0)	0.05	0.08
Factor-B (µg/ml)	262.0 (65.0)	293.0 (55.0)	0.08	0.19
Factor-H (µg/ml)	310.0 (66.0)	358.0 (57.0)	0.01	0.04
Properdin (µg/ml)	27.6 (6.7)	29.9 (7.7)	0.3	0.07

Using the combined group of women (GDM and ND), partial correlations (**Table 3**) and Pearson bivariate analysis (**Supplementary Table 1**) were used to determine correlations between the complement proteins and the demographic, clinical and biochemical data and between members of the complement system (**Table 4** and **Supplementary Table 2**).

**Table 3:** Partial correlations of complement C3, complement C3b/iC3b, complement 4, and Factor-H with demographic and biochemical data for the combined cohort (control and GDM).

	Complement C3		Complement C3b/iC3b		Complement C4		Factor-H	
	r	p	r	p	r	p	r	p
Systolic Blood Pressure baseline (mmHg)	0.17	0.26	−002	0.90	−0.02	0.87	0.13	0.37
Diastolic Blood Pressure baseline (mmHg)	−0.087	0.58	−0.06	0.70	−0.13	0.37	−0.13	0.37
GDM diagnosis/gestational age (weeks)	−0.027	0.89	−0.04	0.81	0.04	0.80	−0.02	0.89
Plasma glucose (mmol/L)	0.17	0.39	−0.006	0.97	0.18	0.35	0.14	0.47
Insulin (uIU/L)	−0.07	0.65	−0.18	0.23	−0.17	0.25	0.02	0.89
Glycated hemoglobin (HbA1c) (%)	0.002	0.99	0.06	0.70	0.25	0.11	0.30	0.05
Alanine aminotransferase (U/l)	−0.03	0.85	0.55	0.73	0.37	0.02	0.12	0.45
Aspartate transaminase (U/l)	−0.02	0.88	0.07	0.67	0.22	0.16	0.10	0.55
Cholesterol (mmol/l)	−0.04	0.85	−0.03	0.88	0.12	0.55	0.24	0.23
Triglycerides (mmol/l)	0.21	0.31	−0.04	0.85	0.10	0.62	−0.05	0.80
High density lipoproteins (mmol/l)	−0.21	0.30	−0.02	0.92	−0.09	0.67	−0.12	0.57
Low density lipoproteins (mmol/l)	−0.05	0.79	−0.02	0.93	0.16	0.44	0.38	0.05
C-reactive protein (mg/ml)	0.24	0.12	0.19	0.21	0.24	0.12	0.30	0.048
2nd Term Systolic Blood Pressure (mmHg)	−0.14	0.36	−0.26	0.08	−0.26	0.09	−0.10	0.50
2nd Term Diastolic Blood Pressure (mmHg)	−0.12	0.42	−0.14	0.36	0.31	0.038	0.24	0.11
Weight at delivery (kg)	−0.06	0.68	−003	0.85	−0.001	0.99	−0.07	0.66
Gestational Age at delivery (weeks)	−0.19	0.20	0.05	0.76	−0.17	0.25	−0.14	0.34
Baby Weight (grams)	−0.26	0.09	−0.14	0.37	−0.10	0.52	−0.25	0.11

**Table 4:** Partial correlations of complement C3, complement C3b/iC3b, complement 4, and Factor-H with members of the complement system from the combined cohort (control and GDM) adjusted for age and BMI.

	Complement C-3		Complement C3b/iC3b		Complement C4		Factor-H	
	r	p	r	p	r	p	r	p
Anti-complement C2	0.33	0.03	0.57	<0.0001	0.43	0.002	0.45	0.001
Anti-Complement C4b	0.16	0.28	0.27	0.07	0.80	<0.0001	0.32	0.03
Anti-Complement C5	0.23	0.11	0.26	0.07	0.46	0.001	0.56	<0.0001
Anti-Complement C5a	0.15	0.30	0.55	<0.0001	0.35	0.02	0.14	0.34
Anti-complement Factor-D	−0.20	0.18	−0.24	0.11	−0.007	0.96	0.02	0.90
Anti-Mannose-binding lectin	0.04	0.79	−0.11	0.47	0.15	0.30	0.19	0.20
Anti-complement factor-1	0.27	0.06	0.47	0.001	0.38	0.009	0.32	0.03
Complement- C1q	0.21	0.16	0.25	0.09	0.47	0.0008	0.61	<0.0001
Complement C-3	1.00		0.61	<0.0001	0.33	0.02	0.40	0.005
Complement C3b/iC3b	0.61	<0.0001	1.00		0.51	0.0002	0.43	0.002
Complement C4	0.33	0.02	0.51	0.0002	1.00		0.68	<0.0001
Factor-B	0.37	0.009	0.42	0.003	0.56	<0.0001	0.76	<0.0001
Factor-H	0.40	0.005	0.43	0.002	0.68	<0.0001	1.000	
Properdin	0.28	0.054	0.26	0.08	0.44	0.002	0.62	<0.0001

Using partial correlations, a correlation was found between complement C4 and ALT (r=0.37, p=0.02) and second trimester diastolic blood pressure (r=0.31, p=0.038); Factor-H correlated with CRP (r=0.30, p=0.048) (**Table 3**).

Using Pearson bivariate analysis, a positive correlation was found for complement C3 with age (r=0.29, p=0.04), BMI (r=0.30, p=0.03), CRP (r=0.32, p=0.03) while there was a negative correlation with gestational age at delivery (r=-0.29, p=0.047) (**Supplementary Table 1**). Complement C4 correlated positively with ALT (r=0.40, p=0.008), CRP (r=0.35, p=0.02) and second trimester diastolic blood pressure (DBP) (r=0.29, p=0.04). Factor-H correlated positively with HbA1c (r=0.32, p=0.03) and CRP (r=0.41, p=0.005) (**Supplementary Table 1**).

Multiple correlations were found between complement C3, complement C4 and Factor-H and other complement and anti-complement proteins, reflecting their closely integrated regulation (**Table 4** and **Supplementary Table 2**).

Regression analysis of all women combined showed that the elevation of C3 could be accounted for by age, BMI, GA, and CRP, with CRP being the most important predictor (p=0.024). Complement C3b/iC3b could be accounted for by second trimester SBP. C4 could be accounted for by ALT, CRP, and SBP. CRP predicted Factor-H elevation.

## Discussion

The results of this study show an elevation of Complement C3, C4 and Factor-H in GDM versus ND control pregnant women in the second trimester of pregnancy. Of note, the elevation in complement factors in GDM over that of normal pregnancy could largely be accounted for by inflammation, as assessed by CRP, suggesting that C3, C4 and Factor-H are not independently associated with GDM.

Complement C3 has been shown to increase with normal pregnancy (18) and fall at term (30), but their levels in the second trimester have not been described. Also, C3 has been shown to be further elevated in pre-eclampsia (24). Furthermore, increased C3 is associated with obesity, dyslipidaemia, inflammation, insulin resistance and liver dysfunction (32), most of the known factors associated with an increased risk of preeclampsia (33). Many of these features were seen in the women with GDM who were more obese, older, and with higher ALT levels. CRP was the greatest predictor of an elevated C3 and, when taken into account together with age and BMI in the regression model, then C3 was no longer significantly elevated, suggesting that it is reflecting the GDM rather than being independently associated with it. Consistent with our data, increased C3 and CRP were associated with preterm delivery in other studies (34, 35).

Complement C4 was also elevated in GDM and associated with CRP as a marker of inflammation but, in the regression model accounting for CRP, ALT and SBP, then C4 was no longer significant, mirroring the findings with C3. ALT levels were higher in GDM, but not above the upper limit of the reference range; however, alterations in ALT are associated with non-alcoholic fatty liver disease which features an increase in insulin resistance, and an elevated ALT is associated with increased risk for GDM (36) though, notably, gamma-glutamyl transferase is a superior predictor of GDM (37). The correlation of C4 with ALT in GDM has not previously been reported.

C4 is associated with systolic blood pressure, and the complement system is associated with arterial hypertension and hypertensive end organ damage (38); however, when adjusted for SBP, then these proteins were not significantly different, suggesting that complement activation was not driving the changes in blood pressure.

Factor H is a serum glycoprotein that accelerates the decay of C3 convertase and is a cofactor for the inactivation of C3b (25). In the absence of factor H, spontaneous activation of the alternative pathway results (23). Factor H, in circumstances of cell stress such as oxidative stress and hypoxia, may be downregulated (39) and that, in turn, would contribute to any underlying inflammatory process (40). Theoretically, the tendency toward increased inflammation in GDM, as seen by the trend toward CRP elevation, is mainly responsible for the increase in C3 found in GDM. This is reflected in the increase in the protective factor H, that correlated with CRP. Increased oxidative stress as occurs in GDM, consequent upon the increase in insulin resistance (27), may reduce factor H and pathologically increase C3 levels.

To our knowledge this is the first study to investigate all the three complement system cascades (classical, alternative and lectin pathway) during pregnancy in GDM subjects. Limitations of this study include the relatively small numbers of pregnant women in each group, which may have prevented the detection of differences between groups. As a cross-sectional study with serum analysis at a single time point during the second trimester, dynamic changes in complement system proteins levels throughout pregnancy could not be assessed. Further, the study was undertaken in a single homogenous population and, while likely generalizable, these findings should be confirmed in other ethnic populations. Further studies on a suitably powered cohort of women with GDM with and without metformin therapy would address the question whether therapy with metformin could have any additional impact on the inflammatory/complement proteins.

## Conclusion

In conclusion, inflammation and increased BMI associated with GDM are likely responsible for the increased C3, C4, and Factor-H seen in the second trimester of pregnancy in GDM that are not independently associated with GDM, and the elevation of C3 was negatively associated with GA; further work is needed to determine if this is predictive.

## Data Availability Statement

The raw data supporting the conclusions of this article will be made available by the authors.

## Ethics Statement

The studies involving human participants were reviewed and approved by Institutional Review Boards of the Hamad Medical Corporation, Qatar (15101/15) and Weill Cornell Medicine-Qatar (15-00016). The patients/ participants provided their written informed consent to participate in this study.

## Author Contributions

MR, MA, and JJ performed the complement protein measurements and contributed to the manuscript. MR, LA, MA, AM, ME, and A-BA-S helped with data analysis, preparation of tables, and contributed to manuscript preparation. AB researched the data and wrote the manuscript. SH helped with statistical analysis. AG captured and provided hormonal and biochemistry data. AM researched data and contributed to manuscript preparation. MB and SA were involved in study design, sample collection and data analysis. MR, SA, and A-BA-S designed the experiments, supervised progress, analyzed data, and revised and approved the final version of the article. All authors contributed to the article and approved the submitted version.

## Funding

MR is supported by Qatar Metabolic Institute and this study was supported by Qatar Metabolic Institute, Hamad Medical Corporation.

## Conflict of Interest

The authors declare that the research was conducted in the absence of any commercial or financial relationships that could be construed as a potential conflict of interest.

## Supplementary Material

Supplementary Table 1 Pearson correlations of complement C3, complement C3b/iC3b, complement 4 and Factor-H with demographic and biochemical data for the combined cohort (control and GDM).

Supplementary Table 2 Pearson correlations of complement C3, complement C3b/iC3b, Complement 4 and Factor-H with members of the complement system from the combined cohort (control and GDM).
