# Corrigendum: Association of Complement-Related Proteins in Subjects With and Without Second Trimester Gestational Diabetes

**DOI:** 10.3389/fendo.2021.781347

**Published:** 2021-11-09

**Authors:** Manjunath Ramanjaneya, Alexandra E. Butler, Meis Alkasem, Mohammed Bashir, Jayakumar Jerobin, Angela Godwin, Abu Saleh Md Moin, Lina Ahmed, Mohamed A. Elrayess, Steven C. Hunt, Stephen L. Atkin, Abdul-Badi Abou-Samra

**Affiliations:** ^1^ Qatar Metabolic Institute, Hamad Medical Corporation, Doha, Qatar; ^2^ Translational Research Institute, Hamad Medical Corporation, Doha, Qatar; ^3^ Diabetes Research Center (DRC), Qatar Biomedical Research Institute (QBRI), Hamad Bin Khalifa University (HBKU), Qatar Foundation (QF), Doha, Qatar; ^4^ Department of Laboratory Medicine and Pathology, Qatar Rehabilitation Institute, Hamad Medical Corporation, Doha, Qatar; ^5^ Department of Genetic Medicine, Weill Cornell Medicine-Qatar, Doha, Qatar; ^6^ Biomedical Research Center (BRC), Qatar University, Doha, Qatar; ^7^ Post Graduate Studies and Research, Royal College of Surgeons in Ireland Bahrain, Adliya, Bahrain

**Keywords:** complement, pregnancy, gestational diabetes, type 2 diabetes, preterm delivery

In the original article, there was an error. One of the funders was missed out in the Acknowledgements.

A correction has been made to the Acknowledgements section.

“The authors would like to thank Qatar Metabolic Institute, Medical Research Center, Translational Research Institute, Hamad Medical Corporation, Doha, Qatar for supporting the study. And Medical Research Center, Hamad Medical Corporation for the article processing fees support”.

The authors apologize for this error and state that this does not change the scientific conclusions of the article in any way. The original article has been updated.

## Publisher’s Note

All claims expressed in this article are solely those of the authors and do not necessarily represent those of their affiliated organizations, or those of the publisher, the editors and the reviewers. Any product that may be evaluated in this article, or claim that may be made by its manufacturer, is not guaranteed or endorsed by the publisher.

