# Supplementary material for: Association of Complement-Related Proteins in Subjects With and Without Second Trimester Gestational Diabetes
Source: Front Endocrinol (Lausanne). 2021 Mar 30;12:641361. doi: 10.3389/fendo.2021.641361 (PMC8043150; doi:10.3389/fendo.2021.641361)
Supplement: Supplementary Table 1 — Pearson correlations of complement C3, complement C3b/iC3b, complement 4 and Factor-H with demographic and biochemical data for the combined cohort (control and GDM). [file Table_1.docx]

**Supplementary Table 1.** Pearson correlations of complement C3, complement C3b/iC3b, complement 4 and Factor-H with demographic and biochemical data for the combined cohort (control and GDM).

|  | Complement C3 | | Complement C3b/iC3b | | Complement C4 | | Factor-H | |
| --- | --- | --- | --- | --- | --- | --- | --- | --- |
|  | r | p | r | p | r | p | r | p |
| Age (years) | 0.29^*^ | 0.044 | 0.20 | 0.17 | 0.08 | 0.59 | 0.13 | 0.38 |
| BMI (kg/m2) | 0.30^*^ | 0.03 | 0.06 | 0.67 | 0.15 | 0.29 | 0.18 | 0.21 |
| Systolic Blood Pressure baseline (mmHg) | 0.26 | 0.06 | 0.02 | 0.94 | 0.04 | 0.79 | 0.19 | 0.18 |
| Diastolic Blood Pressure baseline (mmHg) | -0.11 | 0.44 | -0.10 | 0.51 | -0.13 | 0.37 | -0.14 | 0.34 |
| GDM diagnosis/gestational age (weeks) | 0.06 | 0.67 | -0.02 | 0.86 | 0.08 | 0.59 | 0.03 | 0.84 |
| Plasma glucose (mmol/L) | 0.29 | 0.12 | 0.048 | 0.80 | 0.22 | 0.24 | 0.18 | 0.35 |
| Insulin (uIU/L) | 0.04 | 0.82 | -0.11 | 0.45 | -0.15 | 0.32 | 0.05 | 0.73 |
| Glycated hemoglobin (HbA1c) (%) | 0.08 | 0.60 | 0.12 | 0.42 | 0.26 | 0.08 | 0.32^*^ | 0.03 |
| Alanine aminotransferase (U/l) | 0.05 | 0.77 | 0.08 | 0.63 | 0.40^**^ | 0.008 | 0.17 | 0.28 |
| Aspartate transaminase (U/l) | -0.003 | 0.99 | 0.07 | 0.63 | 0.23 | 0.14 | 0.10 | 0.50 |
| Cholesterol (mmol/l) | -0.03 | 0.87 | -0.002 | 0.99 | 0.09 | 0.65 | 0.21 | 0.28 |
| Triglycerides (mmol/l) | 0.21 | 0.28 | 0.03 | 0.90 | 0.08 | 0.70 | -0.08 | 0.67 |
| High density lipoprotein (mmol/l) | -0.22 | 0.26 | -0.03 | 0.89 | -0.11 | 0.58 | -0.14 | 0.46 |
| Low density lipoprotein (mmol/l) | -0.07 | 0.71 | -0.008 | 0.97 | 0.13 | 0.51 | 0.34 | 0.08 |
| C-reactive protein (mg/ml) | 0.32^*^ | 0.03 | 0.19 | 0.20 | 0.35^*^ | 0.02 | 0.41^**^ | 0.005 |
| 2nd Term Systolic Blood Pressure (mmHg) | -0.14 | 0.35 | -0.26^*^ | 0.07 | -0.25 | 0.08 | -0.10 | 0.48 |
| 2nd Term Diastolic Blood Pressure (mmHg) | -0.10 | 0.48 | -0.11 | 0.44 | 0.29^*^ | 0.044 | 0.22 | 0.13 |
| Weight at delivery (kg) | 0.17 | 0.24 | -0.003 | 0.99 | 0.15 | 0.31 | 0.13 | 0.36 |
| Gestational Age at delivery (weeks) | -0.29^*^ | 0.047 | 0.001 | 0.99 | -0.23 | 0.12 | -0.21 | 0.14 |
| Baby Weight (grams) | -0.14 | 0.36 | 0.11 | 0.49 | -0.09 | 0.56 | -0.16 | 0.13 |

**Supplementary Table 2.** Pearson correlations of complement C3, complement C3b/iC3b, Complement 4 and Factor-H with

members of the complement system from the combined cohort (control and GDM).

|  | Complement C-3 | | Complement C3b/iC3b | | Complement C4 | | Factor-H |  |
| --- | --- | --- | --- | --- | --- | --- | --- | --- |
|  | r | p | r | p | r | p | r | p |
| Anti-complement C2 | 0.28 | 0.11 | 0.53** | 0.0001 | 0.39** | 0.005 | 0.40** | 0.004 |
| Anti-Complement C4b | 0.11 | 0.45 | 0.20 | 0.18 | 0.77** | 0.000 | 0.30* | 0.036 |
| Anti-Complement C5 | 0.17 | 0.24 | 0.19 | 0.20 | 0.44** | 0.002 | 0.52** | 0.0001 |
| Anti-Complement C5a | 0.11 | 0.47 | 0.51** | 0.000 | 0.334* | 0.02 | 0.12 | 0.40 |
| Anti-complement Factor-D | -0.18 | 0.22 | -0.28 | 0.06 | 0.01 | 0.96 | 0.03 | 0.86 |
| Anti-Mannose-binding lectin | 0.03 | 0.86 | -0.12 | 0.43 | 0.14 | 0.34 | 0.18 | 0.22 |
| Anti-complement factor-1 | 0.25 | 0.09 | 0.45** | 0.001 | 0.36* | 0.011 | 0.31* | 0.03 |
| Complement- C1q | 0.18 | 0.22 | 0.26 | 0.07 | 0.43** | 0.002 | 0.56** | 0.000 |
| Complement C-3 | 1.000 |  | 0.61** | 0.000 | 0.35* | 0.012 | 0.44** | 0.002 |
| Complement C3b/iC3b | 0.61** | 0.0001 | 1.000 |  | 0.50** | 0.0001 | 0.45** | 0.001 |
| Complement C4 | 0.35* | 0.012 | 0.50** | 0.0001 | 1.000 |  | 0.69** | 0.0001 |
| Factor-B | 0.40** | 0.004 | 0.40** | 0.004 | 0.57** | 0.0001 | 0.76** | 0.0001 |
| Factor-H | 0.44** | 0.002 | 0.45** | 0.001 | 0.69** | 0.0001 | 1.000 |  |
| Properdin | 0.33* | 0.02 | 0.32* | 0.03 | 0.42** | 0.002 | 0.60** | 0.0001 |
